# Editorial: Digital Health Literacy and Black Older Adults: Bridging the Digital Divide in Managing Chronic Conditions

**DOI:** 10.1002/nop2.70123

**Published:** 2024-12-15

**Authors:** Brenda Owusu, Serina Gbaba, Judith Juste, Balkys Bivins, Diana Baptiste

**Affiliations:** ^1^ School of Nursing and Health Sciences University of Miami Coral Gables Florida USA; ^2^ Johns Hopkins School of Nursing Baltimore Maryland USA

In an increasingly digital world, technology has become an essential tool for managing health, particularly for individuals living with chronic conditions like diabetes and heart disease. Yet, for many older adults, especially those from underserved communities, the promise of these advancements remains limited. In the United States, health disparities are often more pronounced among Black older adults due to factors such as a lack of trust in healthcare systems, low health literacy and socio‐economic challenges (Tappen et al. 2022). Among Black older adults, the digital divide, which is a gap in access to and use of technology, presents a significant barrier to achieving health equity. Digital literacy refers to ‘the ability to use information and communication technologies to find, evaluate, create, and communicate information, requiring both cognitive and technical skills’ (van Kessel et al. 2022). For patients to effectively use telehealth technologies, they must be proficient in accessing and utilising digital tools appropriately for healthcare purposes. Digital and health literacy, although two separate concepts, interact within the healthcare context, specifically among vulnerable persons. In this editorial, we discuss the gaps in digital health literacy among Black older adults, contributing to a digital divide.

As healthcare providers continue adopting telehealth and mobile health technologies to improve health management, it is crucial to address the unique challenges these Black older adults face. Without intentional efforts to enhance the digital literacy of older Black adults and their access to technology, we risk leaving behind some of the most vulnerable members of our society (Tappen et al. 2022; van Kessel et al. 2022). Furthermore, limited digital health literacy represents a broader context of social determinants of health (van Kessel et al. 2022). The philosophy of John Rawls emphasises fairness and equitable justice, which is in alignment with addressing the digital divide for older Black adults. His social justice theory takes this notion beyond healthcare being a fundamental human right and addresses health equity and social justice being vitalto preserve lives (Rawls 2005).

Recent research has highlighted the importance of improving digital health literacy to enhance self‐management among Black older adults with chronic conditions, emphasising that disparities in technology use can lead to worsened health outcomes (Czaja et al. 2018; Tappen et al. 2022; Williams and Shang 2024). Even with advancements that have made technological devices more user‐friendly for Black older adults, the digital divide persists. Many older adults still face barriers that hinder their ability to use new technology effectively and are less likely to embrace these innovations than younger generations (Williams and Shang 2024). Addressing these barriers is critical in ensuring equitable healthcare access for all populations.

## 1 Bridging the Digital Divide

To effectively bridge the digital divide for older adults, particularly among marginalised populations, it is imperative to foster collaborative efforts among healthcare providers, policymakers and community leaders. These stakeholders must work together to develop and implement strategies that enhance access to technology, improve digital literacy and ensure equitable healthcare delivery (Williams and Shang 2024). Evidence‐based recommendations for enhancing digital literacy offer a promising approach to achieving sustainable improvements in chronic disease management among Black older adults (Williams and Shang 2024). Healthcare providers play a crucial role in identifying the barriers their patients face and advocating for solutions tailored to the needs of older adults. Policymakers must prioritise legislation that expands coverage for telehealth services and digital health tools, addressing gaps in Medicare and promoting infrastructure development in underserved areas (Williams and Shang 2024). Community leaders can also facilitate outreach programmes that educate Black older adults on using technology and connect them with available resources (Tappen et al. 2022; Williams and Shang 2024).

By aligning efforts across these sectors, we can create a comprehensive support system that empowers older adults to engage in their healthcare and leverage technology for better health outcomes. Such collaboration is essential for addressing health disparities and promoting health equity in our society (van Kessel et al. 2022; Williams and Shang 2024).

## 2 Implications for Nursing

The role of nurses is crucial in addressing the barriers older adults face in accessing and utilising technology for health management, particularly among those with chronic conditions such as diabetes and heart disease. One of the key responsibilities of nurses is advocacy for digital literacy programmes (Williams and Shang 2024). By participating in and promoting workshops culturally tailored to Black older adults, nurses can facilitate the development of digital literacy initiatives that help bridge the digital divide. This involvement is essential in equipping older adults with the necessary skills to effectively navigate technology for their health management (Tappen et al. 2022).

Additionally, nurses must conduct a thorough assessment of technology barriers. This involves evaluating patients' access to technology and identifying specific challenges Black older adults encounter in utilising digital health tools. Factors such as internet connectivity, device familiarity and overall health literacy should be considered. Understanding these barriers enables nurses to customise their care strategies and provide individualised support to their patients (Czaja et al. 2018; National Institute on Aging 2021). The integration of technology into care plans is another vital aspect of nursing practice. By incorporating telehealth services and mobile health applications, nurses can enhance the self‐management of chronic conditions, improve communication and monitoring of patients' health. Such innovations not only make healthcare more accessible but also empower older adults to take an active role in their health management (National Institute on Aging 2021).

Furthermore, it is crucial for nurses to provide education, inform patients about digital health resources, including telehealth options and community support for technology use and empower Black older adults to utilise these tools effectively, ultimately leading to better health outcomes (Czaja et al. 2018). Finally, continuous professional development is vital for nurses to remain abreast of technological advancements in healthcare. By understanding new tools and their applications, nurses can offer informed guidance and support to older adults, facilitating their adaptation to the evolving landscape of digital health (Tappen et al. 2022; National Institute on Aging 2021). Embracing these roles, nurses can significantly contribute to bridging the digital divide for older adults, ensuring equitable healthcare access and improved health outcomes for some of the most vulnerable members of our society.

## 3 Conclusion

The digital divide among Black older adults remains a significant barrier to achieving health equity and hinders the provision of quality healthcare access to this vulnerable population. Nurses are well positioned to address this gap and promote greater technological inclusion. To achieve meaningful progress, nurses must collaborate with multidisciplinary teams, including healthcare providers, policymakers and community leaders, to advocate for initiatives that foster digital literacy. Targeted interventions, such as culturally relevant workshops and individualised support programmes, can empower Black older adults by equipping them with the necessary skills to navigate digital platforms effectively.

Improving digital competency among Black older adults can potentially enhance chronic disease self‐management, promote better access to telehealth and other online resources and ultimately reduce morbidity and mortality in this population. Additionally, prioritising policies that fund community‐based digital literacy programmes and expand access to affordable technology is essential for ensuring sustainable progress. By addressing these structural and educational challenges, healthcare systems can become more inclusive, enabling Black older adults to actively engage in their care and improve health outcomes over the long term.

## Conflicts of Interest

The authors declare no conflicts of interest.

## Data Availability

This study did not generate any new data. All data supporting the findings of this editorial were derived from previously published sources, which are appropriately cited within the text.
